# Dosimetric evaluation of synthetic CT relative to bulk density assignment-based magnetic resonance-only approaches for prostate radiotherapy

**DOI:** 10.1186/s13014-015-0549-7

**Published:** 2015-11-24

**Authors:** Joshua Kim, Kim Garbarino, Lonni Schultz, Kenneth Levin, Benjamin Movsas, M. Salim Siddiqui, Indrin J. Chetty, Carri Glide-Hurst

**Affiliations:** Department of Radiation Oncology, Henry Ford Health System, 2799 W. Grand Blvd, Detroit, MI 48202 USA

**Keywords:** MR simulation, Synthetic CT, Radiotherapy treatment planning, Radiation oncology

## Abstract

**Background:**

Magnetic resonance imaging (MRI) has been incorporated as an adjunct to CT to take advantage of its excellent soft tissue contrast for contouring. MR-only treatment planning approaches have been developed to avoid errors introduced during the MR-CT registration process. The purpose of this study is to evaluate calculated dose distributions after incorporating a novel synthetic CT (synCT) derived from magnetic resonance simulation images into prostate cancer treatment planning and to compare dose distributions calculated using three previously published MR-only treatment planning methodologies.

**Methods:**

An IRB-approved retrospective study evaluated 15 prostate cancer patients that underwent IMRT (*n* = 11) or arc therapy (*n* = 4) to a total dose of 70.2-79.2 Gy. Original treatment plans were derived from CT simulation images (CT-SIM). T1-weighted, T2-weighted, and balanced turbo field echo images were acquired on a 1.0 T high field open MR simulator with patients immobilized in treatment position. Four MR-derived images were studied: bulk density assignment (10 HU) to water (MR_W_), bulk density assignments to water and bone with pelvic bone values derived either from literature (491 HU, MR_W+B491_) or from CT-SIM population average bone values (300 HU, MR_W+B300_), and synCTs. Plans were recalculated using fixed monitor units, plan dosimetry was evaluated, and local dose differences were characterized using gamma analysis (1 %/1 mm dose difference/distance to agreement).

**Results:**

While synCT provided closest agreement to CT-SIM for D95, D99, and mean dose (<0.7 Gy (1 %)) compared to MR_W,_ MR_W+B491_, and MR_W+B300_, pairwise comparisons showed differences were not significant (*p* < 0.05). Significant improvements were observed for synCT in the bladder, but not for rectum or penile bulb. SynCT gamma analysis pass rates (97.2 %) evaluated at 1 %/1 mm exceeded those from MR_W_ (94.7 %), MR_W+B300_ (94.0 %), or MR_W+B491_ (90.4 %). One subject’s synCT gamma (1 %/1 mm) results (89.9 %) were lower than MR_W_ (98.7 %) and MR_W+B300_ (96.7 %) due to increased rectal gas during MR-simulation that did not affect bulk density assignment-based calculations but was reflected in higher rectal doses for synCT.

**Conclusions:**

SynCT values provided closest dosimetric and gamma analysis agreement to CT-SIM compared to bulk density assignment-based CT surrogates. SynCTs may provide additional clinical value in treatment sites with greater air-to-soft tissue ratio.

## Background

Radiation therapy treatment planning was developed using computed tomography (CT) as its base imaging modality due to accurate geometric fidelity and the straightforward conversion from linear attenuation coefficients to electron density values. However, a major drawback in CT is its poor soft tissue contrast that makes it difficult to accurately identify and contour soft tissue structures. Conversely, magnetic resonance imaging (MRI) provides excellent soft tissue contrast. Thus, efforts have been made to incorporate MRI into the treatment planning process. MR images would first be registered to the CT simulation image (CT-SIM), and MR-contoured structures would then be transferred onto the CT-SIM for treatment planning. Unfortunately, the registration process introduces additional systematic uncertainties (~1-2 mm for pelvis) [1] that would propagate throughout the treatment planning workflow. Moreover, having two simulation modalities can be cost-prohibitive while introducing additional burden on the clinical workflow. Therefore, interest has grown in developing an MR-only workflow for radiotherapy treatment planning [2, 3].

While the benefits of MR imaging (i.e. excellent soft tissue contrast and lack of ionizing radiation) are substantial, several logistical difficulties must be overcome before MR-only simulation for radiotherapy would be practical clinically. The first major issue is geometric distortion, which is typically categorized as either machine-specific or patient-specific. The main machine-specific sources of distortion are inhomogeneity of the main magnetic field and non-linearity in the magnetic field produced by gradient coils, while magnetic susceptibility-induced distortion is the dominant form of patient-specific errors. Many papers have been published characterizing the magnitude of these distortions and the post-processing of the image needed to correct for them [4]. Through various correction schemes and the use of MR sequences such as 3D turbo spin echo sequences, it has been reported that distortions can be reduced to within acceptable tolerance for radiotherapy purposes for many treatment sites [3].

Another significant limitation of MRI is that there is no fundamental relationship between MRI image intensity values and the electron density information required by treatment planning systems to accurately account for heterogeneity within a patient for accurate dose calculation. The most straightforward method to overcome this obstacle is to assume no significant heterogeneities exist and assign a bulk electron density value equivalent to water within the entire body contour [5, 6]. However, this approach introduces uncertainties in the prostate of up to 2.5 % [5]. Variations of this method have been introduced where one or more structures that introduce large heterogeneities (e.g. bone or air) are segmented and assigned appropriate relative electron density values [3, 6, 7], which reduced the dose uncertainty to less than 1.5 %. Other methods that have been introduced are based on the use of atlases [8] or statistical models [9, 10]. Atlas-based methods may struggle with atypical anatomy, such as cases with local recurrence after radical prostatectomy. Presently, studies incorporating statistical methods have focused mainly in the brain region and rely on sequences that may have difficulty with larger field of view (FOV) body sites such as the pelvis.

We recently introduced a novel, voxel-based, weighted-summation method for generating synthetic CTs (synCTs) from MRI images for male pelvis anatomy [11]. While preliminary dosimetric comparisons were made between synCT and CT-SIM, we build on our initial investigation by evaluating synCT performance relative to three other established approaches for MR-only treatment planning of prostate cancer. In this paper, the use of synCT for dose calculation was compared to the use of bulk density assignment methods in order to elucidate situations where implementation of the synCT algorithm may be beneficial.

## Methods

### Patients

Retrospective analysis was performed for 15 early stage (T1 or T2, N0, M0) prostate cancer patients with a median patient age of 71 years (range: 53–96) that were enrolled in an institutional review board-approved study wherein MR-simulation was performed as an adjunct to the CT-simulation process. The planning target volume (PTV) was composed of the prostate and proximal seminal vesicles for 13 patients, while the two remaining patients were treated for local recurrence in the prostate/seminal vesicle bed after radical prostatectomy. All patients underwent radiotherapy delivered using either intensity-modulated radiation therapy (IMRT, *n* = 11) or volume-modulated arc therapy (VMAT, *n* = 4) to a total dose of 70.2-79.2 Gy. All patients received the same preparation instructions for both imaging sessions: full bladder and empty rectum, which is consistent with our clinical practice [12].

### CT acquisition

A Brilliance Big Bore (Philips Health Care, Cleveland, OH) CT scanner was used to acquire pelvis images using the following parameters: 140 kVp, 500 mAs, 512x512 in-plane image dimensions, 1.28x1.28 mm^2^ in-plane spatial resolution, and 3 mm slice thickness.

### MR acquisition

A 1.0 T Panorama High Field Open (Philips Medical Systems, Best, Netherlands) MR simulator was used with rigid, solenoid-based body coils and a flat tabletop insert (Civco, Orange City, IA). Patients were aligned and leveled to their tattoos from CT simulation, and the same immobilization devices (banded feet and shaped foam pad for legs) were used. T1-weighted fast field echo, T2-weighted turbo spin echo, and balanced turbo field echo images were acquired for each patient.

Sequences were collected using the parameters for repetition time (TR), echo time (TE), flip angle (α), FOV, and image resolution provided in Table 1. Total scan time to collect these sequences was approximately 18 min. An inverse T1 image was also generated by subtracting the T1 image from the intensity value below which fell 95 % of the area under the curve of the intensity value histogram for the T1 image. Inverse T1 images provided high intensity values in bone regions for the synCT algorithm.

**Table 1:** MR sequence parameters

				Acquisition		Reconstruction	
	TR (ms)	TE (ms)	α (°)	FOV (mm^3^)	Voxel size (mm^3^)	Grid size	Voxel size (mm^3^)
T1 Fast field echo	~17	6.9	25	300x400x250	1.50x1.50x2.5	640x640x250 or 576x576x250	0.65x0.65x2.5
T2 Turbo Spin Echo	~5500	80	90	300x400x250	1.00x1.14x2.5	640x640x250 or 576x576x250	0.65x0.65x2.5
bTFE	5.4	2.7	75	300x400x250	1.50x1.50x2.5	672x672x250 or 432x432x250	0.65x0.65x2.5

### CT surrogate generation

**Fig. 1:**
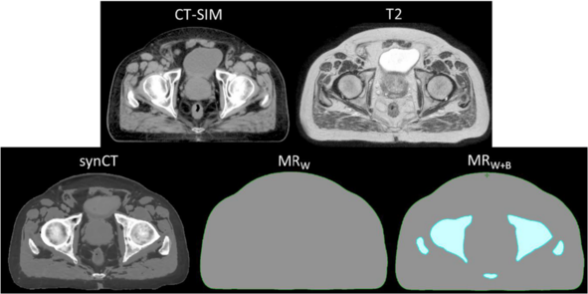
CT and MR-derived CT surrogate images. (Top) Patient CT and T2-weighted MR images. (Bottom) MR-derived CT surrogates used for study: synthetic CT (synCT), T2 with water bulk density assignment (MR_W_), T2 with bulk density assignments of water and bone (MR_W+B_)

Four different established approaches of defining MR-derived CT surrogates were generated for comparison with the CT-SIM. The first was a homogeneous electron density relative to water assignment of 1.0 to the entire external contour (MR_W_) [3]. The second approach, defined as MR (water + bone = 491 HU or MR_W+B491_) included two segments: relative density assignment of 1.0 for the external contour and relative electron density assignment of 1.27 [13] (equivalent to 491 HU) to bone that was manually contoured on the T2-weighted MR image. While assigning 491 HU to bone has been used in the literature, it has been observed that this value may be too high for the femoral heads [14] since the higher proportion of cancellous (i.e. spongy) bone within the femoral head would reduce its relative electron density. Consequently, it has been found that calculating the average CT value within the femoral heads for the patient population provides more accurate relative electron density values that produce dose distributions closer to original CT-based treatment plans [14]. We followed this workflow by contouring the femoral heads for each patient and calculating the average CT value over the full set of 15 patients, which yielded a value of ~300 HU. Therefore, the third approach included a relative density assignment of 1.0 to the external contour as well as an assignment of 300 HU (relative electron density ≈ 1.14) to the femoral heads (water + bone = 300 HU or MR_W+B300_). The final approach was to generate synCTs via the method described in detail in a previous publication [11]. Briefly, manual segmentation of bone was followed by automatic segmentation of four other predetermined classes (air, soft tissue, fat, and fluid) using k-means clustering and morphological operations. Each synCT voxel value was then calculated as a summation of intensity values from the acquired MR images that had been weighted by sequence- and class-specific factors. The various MR-derived surrogates are shown in Fig. 1.

### Treatment planning

All patients were treated using CT-SIM based plans created in the Eclipse® treatment planning system (Varian Medical Systems, Palo Alto, CA) with physician-contoured target and organ-at-risk (OAR) volumes. Treatments were delivered using either IMRT with seven or nine fields delivering 1.8-2.0 Gy/fraction (dose range: 75.6-79.2 Gy) or VMAT using a single arc to deliver 1.8-2.0 Gy/fraction (dose range: 70–78 Gy). Once generated, each CT surrogate was interpolated onto the original CT-SIM image grid and imported into Eclipse®. The synCT was rigidly registered to the CT-SIM image, and all other CT surrogates were registered by applying those same transformation coordinates derived from the registration of the synCT image. Original CT-SIM treatment plans were copied onto each CT surrogate, and dose was recalculated using original plan parameters. Target and OAR contours were then transferred from the CT-SIM to each CT surrogate for analysis.

Standard dose-volume histogram (DVH) metrics were evaluated for the target and OARs. PTVs were compared using D99, D95, and mean dose to prostate where Dx is equal to the dose delivered to x% of the structure volume. Bladders, prostates, and penile bulbs were analyzed using dosimetric indices defined in QUANTEC guidelines [15–17]. Namely, D15, D25, and D35 were calculated for the bladder [15] with observed clinical symptoms as an endpoint. D15, D25, and D35 were calculated for the rectum based on their use in evaluating the risk for late rectal toxicity [16]. Finally, D90 for the penile bulb, which is used to assess the risk of radiotherapy-induced erectile dysfunction [17], was calculated. Absolute differences between dosimetric values calculated using CT-SIM, taken as ground truth, and those calculated using each of the MR-derived CT surrogates were determined. Relative agreement with CT-SIM-derived dose values was evaluated through a paired comparison between each set of pseudo-CT images using nonparametric signed rank tests with the significance level set to *p* = 0.05. Moreover, 2D gamma analysis (evaluated at 1 %/1 mm and 2 %/2 mm dose difference/distance to agreement) was performed to compare axial dose distributions at isocenter for each of the MR-derived treatment plans to those from the CT-SIM-based plans.

## Results

### Dosimetric criteria

**Fig. 2:**
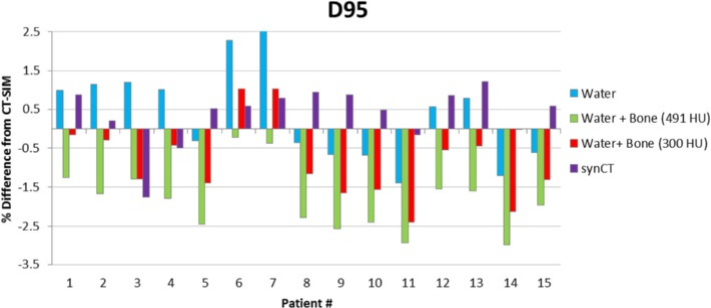
Percent difference in D95 to PTV. Percent difference in D95 to PTV between CT-based dose calculations and dose calculations derived from each CT surrogate for each patient

**Fig. 3:**
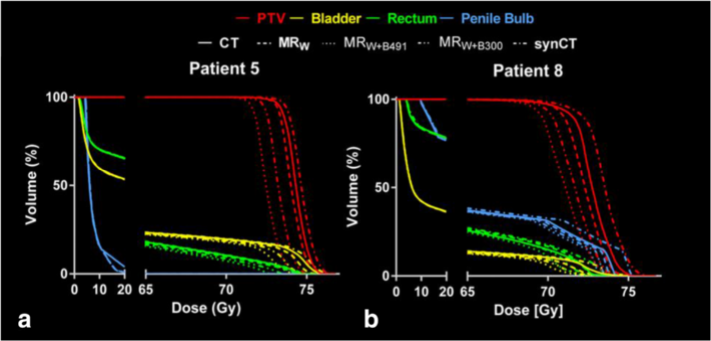
Patient dosimetric results. **a** Average and **b** worst case patient DVHs. Good agreement is observed for all treatment plans. SynCT provided closest agreement to CT-SIM results for PTV and bladder, while no significant differences were observed for rectum and penile bulb

Table 2 compares the population average values along with standard deviation and 95 % confidence intervals for selected DVH metrics calculated using CT-SIM-based treatment plans with those of each CT surrogate-based plans. While synCT yielded the smallest dosimetric differences from CT-SIM for target D95, D99, and mean dose (<0.7 Gy (1 %)) compared to MR_W_, MR_W+B491_, and MR_W+B300_, pairwise comparisons showed that dose reduction was only statistically significant relative to MR_W+B491_. Figure 2 shows that the synCT tended to have closer agreement overall with CT-SIM values, but that patient-specific results were variable. In addition, using population average values for bone assignment (i.e. MR_W+B300_) provided significant improvements relative to MR_W+B491_ for all metrics. For bladder, SynCT-derived dose values displayed closest average agreement with CT-SIM dose values for all metrics, with small (<1 %) but significant improvements relative to MR_W_, MR_W+B491_, and MR_W+B300_. For the rectum and penile bulb, all MR-derived images showed good agreement (difference <1 %) with original CT-SIM calculated values, and no significant differences were observed between synCT, MR_W_, and MR_W+B300_. MR_W+B491_ performed significantly worse for all metrics. Figure 3 displays DVHs for Patient 5, whose OAR results showed average agreement with CT-SIM values, and Patient 8, who displayed the largest deviation from those values (up to 3 %, 2 %, and 3.5 % for bladder, rectum, and penile bulb, respectively). For Patient 8, the bulk density assignment-based approaches produced dose values lower than those produced for CT-SIM because of a higher than normal amount of fatty tissue, where CT-SIM values were lower than bulk assigned HU values. Conversely, while synCT tissue HU values were fairly close to those in CT-SIM, synCT bone HU values tended to be lower for this patient, leading to higher calculated dose values.

**Table 2:** Average and standard deviation results for select target and OAR DVH metrics with 95 % confidence intervals in parentheses

	CT-SIM	MR_W_	MR_W+B491_	MR_W+B300_	synCT
PTV					
D95 (Gy)	74.9 ± 3.5 (72.9, 76.8)	75.2 ± 3.9 (73.2, 77.2)	73.5 ± 3.7 (71.5, 75.6)	74.2 ± 3.8 (72.1, 76.3)	75.1 ± 3.7 (73.2, 77.0)
D99 (Gy)	73.1 ± 3.8 (71.0, 75.2)	73.5 ± 4.1 (71.4, 75.6)	71.8 ± 4.2 (69.4, 74.1)	72.5 ± 4.2 (70.1, 74.8)	73.3 ± 3.8 (71.2, 75.4)
Mean Dose (Gy)	76.8 ± 3.4 (74.9, 78.7)	77.1 ± 3.8 (75.2, 79.1)	75.5 ± 3.6 (73.6, 77.5)	76.2 ± 3.7 (74.2, 78.3)	77.2 ± 3.6 (75.2, 79.2)
Bladder					
D15 (Gy)	65.0 ± 10.4 (59.3, 70.8)	64.9 ± 10.2 (59.3, 70.6)	63.5 ± 10.6 (57.6, 69.4)	64.0 ± 10.8 (58.0, 70.0)	65.0 ± 10.6 (59.1, 70.9)
D25 (Gy)	51.1 ± 10.5 (45.2, 56.9)	50.8 ± 10.4 (45.0, 56.5)	50.0 ± 10.7 (44.0, 55.8)	50.2 ± 10.9 (44.1, 56.2)	51.0 ± 10.5 (45.1, 56.8)
D35 (Gy)	39.1 ± 10.9 (33.1, 45.2)	38.8 ± 10.8 (32.9, 44.8)	38.4 ± 10.8 (32.5, 44.4)	38.6 ± 10.9 (32.5, 44.6)	39.0 ± 10.9 (33.0, 45.1)
Rectum					
D15 (Gy)	64.0 ± 6.0 (60.6, 67.3)	64.2 ± 6.3 (61.0, 67.4)	63.1 ± 6.1 (60.0, 66.1)	63.6 ± 6.2 (60.4, 66.7)	64.3 ± 6.3 (61.0, 68.0)
D25 (Gy)	55.4 ± 6.9 (51.6, 59.2)	55.5 ± 7.0 (51.9, 59.0)	54.7 ± 6.8 (51.3, 58.2)	55.1 ± 6.9 (51.6, 58.6)	55.8 ± 6.8 (52.0, 59.8)
D35 (Gy)	48.8 ± 7.4 (44.7, 52.9)	48.9 ± 7.4 (45.2, 52.6)	48.3 ± 7.3 (44.6, 52.0)	48.6 ± 7.3 (44.9, 52.3)	49.2 ± 7.5 (45.4, 53.0)
Penile Bulb					
D90 (Gy)	13.9 ± 16.9 (4.2, 23.6)	14.9 ± 17.0 (4.9, 24.5)	14.6 ± 16.8 (5.1, 24.7)	14.7 ± 16.9 (5.1, 24.8)	14.8 ± 17.2 (4.7, 24.8)

### Gamma analysis

**Fig. 4:**
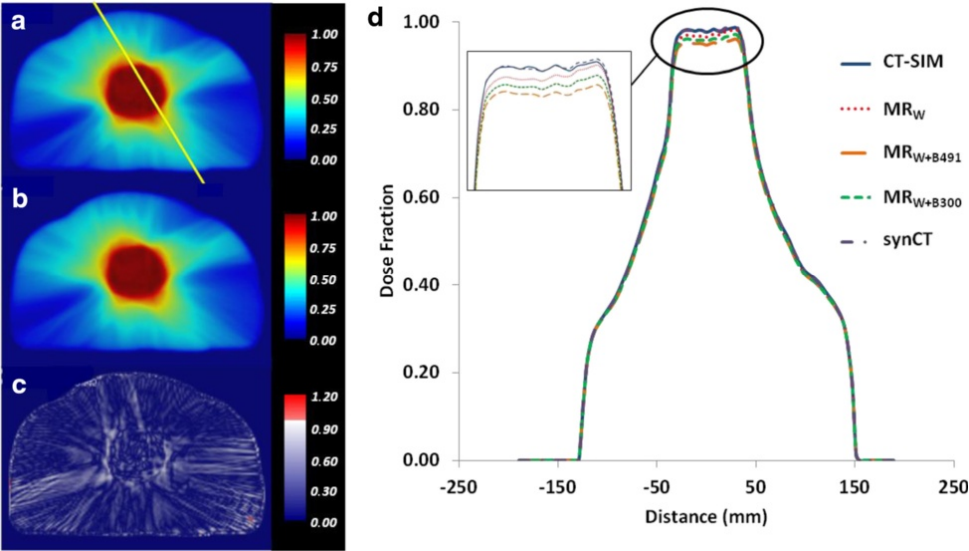
Patient gamma analysis results. Dose plans at isocenter derived from **a** CT-SIM and **b** synCT and resulting **c** gamma distribution (pass rate: 99.95 %) evaluated at 1 %/1 mm for a typical patient (Patient 14) treated to 78 Gy. **d** Line profile through **a** compares dose differences between CT-SIM and CT surrogates

**Fig. 5:**
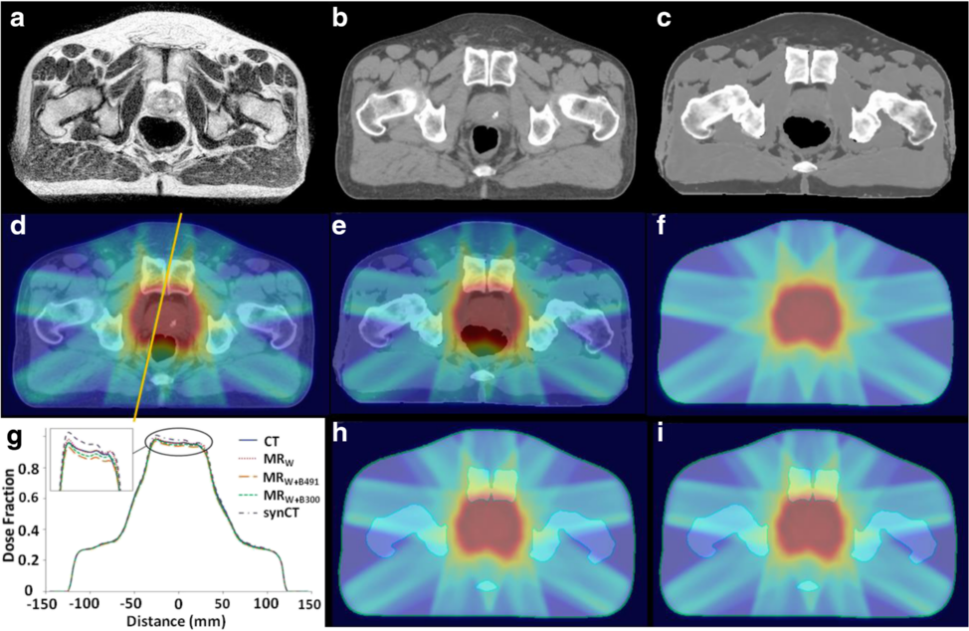
Case study. Visible increase in rectal air volume during MR simulation relative to CT simulation for Patient 11 seen when comparing **a** T2-weighted MR image, **b** CT-SIM, and **c** synCT. Resulting differences in dose distributions at isocenter from **d** CT-SIM were seen in **e** dose distributions from synCT but not from methods assigning bulk electron density values to **f** water, **h** water + bone (491 HU), and **i** water + bone (300 HU). Increased dose for synCT can be seen in **g** vertical line profiles taken through dose planes at isocenter

Overall, synCT gamma analysis pass rates at 1 %/1 mm (97.2 %) exceeded those for MR_W_ (94.7 %), MR_W+B491_ (90.4 %), and MR_W+B300_ (94.0 %) with individual patient results at both 2 %/2 mm and 1 %/1 mm for each CT surrogate shown in Table 3. Generally, gamma analysis conducted at 2 %/2 mm was not sensitive enough to reveal significant differences between CT surrogates (pass rates ranging from 97.8 % (MR_W+B491_) to 99.8 % (synCT)). Figure 4 displays a typical gamma analysis case where the synCT showed slightly better agreement than alternative CT surrogate methods. One exception to this trend occurred for Patient 11, whose gamma analysis results at 1 %/1 mm were lower for synCT (89.9 %) than for MR_W_ (98.7 %) or MR_W+B300_ (96.7 %). As Fig. 5a illustrates, there was a large increase in rectal gas during MR simulation relative to that present during CT simulation. While this did not affect the bulk density assignment methodologies tested, Fig. 5 shows that the change in air volume was propagated to the generated synCT (Fig. 5e). The increased air volume led to higher calculated doses for the synCT-derived treatment plan while the bulk density assignment-based methods remained unaffected (Fig. 5g). For this case, synCT rectal doses were systematically higher than all other approaches: MR_W_ by 1.6-2.6 Gy (4.3-5.6 %), MR_W+B300_ by 1.8-3.0 Gy (4.6-5.8 %), and MR_W+B491_ by 2.0-3.5 Gy (5.0-6.2 %).

**Table 3:** Patient gamma analysis results for both 2 %/2 mm and 1 %/1 mm dose difference/distance to agreement

	MR_W_		MR_W+B491_		MR_W+B300_		synCT	
	2 %/2 mm	1 %/1 mm	2 %/2 mm	1 %/1 mm	2 %/2 mm	1 %/1 mm	2 %/2 mm	1 %/1 mm
Patient 1	1.00	0.96	1.00	0.95	1.00	0.99	1.00	0.98
Patient 2	1.00	0.96	1.00	0.97	1.00	1.00	1.00	1.00
Patient 3	0.97	0.90	1.00	0.99	1.00	0.98	1.00	0.99
Patient 4	1.00	0.98	0.97	0.91	1.00	0.98	1.00	0.97
Patient 5	1.00	0.99	0.96	0.88	1.00	0.93	1.00	0.99
Patient 6	0.93	0.87	1.00	0.99	1.00	0.94	1.00	0.99
Patient 7	0.96	0.91	1.00	0.99	1.00	0.96	1.00	0.99
Patient 8	0.99	0.94	0.93	0.82	0.98	0.88	0.99	0.95
Patient 9	1.00	0.98	0.96	0.91	0.99	0.94	1.00	0.99
Patient 10	1.00	0.99	0.99	0.93	1.00	0.96	1.00	1.00
Patient 11	1.00	0.82	0.96	0.64	0.99	0.72	1.00	0.90
Patient 12	1.00	0.99	0.99	0.90	1.00	0.97	0.99	0.90
Patient 13	1.00	0.97	1.00	0.92	1.00	1.00	1.00	0.94
Patient 14	1.00	0.97	0.94	0.86	0.98	0.92	1.00	1.00
Patient 15	1.00	0.98	0.98	0.90	1.00	0.94	1.00	0.99
Mean	0.99	0.95	0.98	0.90	1.00	0.94	1.00	0.97
Min	0.93	0.82	0.93	0.64	0.98	0.72	0.99	0.90
Max	1.00	0.99	1.00	0.99	1.00	1.00	1.00	1.00

## Discussion

This work sought to incorporate synCTs and previously published bulk density assignment-based CT surrogates into the development of radiotherapy treatment plans and to evaluate the resulting dose distributions in order to determine the impact of integrating synCT into the treatment planning workflow. Overall, target and OAR dose metrics and gamma analysis used for comparison revealed that synCT-derived treatment plans tended to provide closer agreement to CT-SIM than bulk density assignment-based approaches. While 3D gamma analysis has recently emerged as a tool for evaluating the entire irradiated volume of both target and OARs, 2D gamma analysis has been reported to yield more stringent results [18] and is consistent with our clinical practice. The most significant OAR differences were observed for the bladder. Consistent with the literature [14], the MR_W+B491_ tended to perform significantly worse than other MR-derived images due to its high bone relative electron density values that yielded slight underestimations of dose to target and OARs relative to the CT-SIM plan. Use of population average-derived relative electron density values for MR_W+B300_ provided better agreement with original CT-SIM plan dose distributions than MR_W+B491_. However, the improvement, though statistically significant, was minimal (~0.5 %), which is consistent with previous reports (~0.6 %) [14].

Common external contours were used that may mask dosimetric differences between dose distributions calculated using CT-SIM and those using MR-derived CT substitutes due to changes in treatment beam path length. These were added to correct for changes in patient geometry between different patient setups, particularly for cases having a time lapse of up to two weeks between CT and MR simulation. This effect was assumed to be small. Consistent with that assumption, the number of added voxels amounted to <5 % of the whole, and these were mainly concentrated around the abdomen and out of the path of the treatment beams.

With the exception of MR_W_, which does not take into account tissue heterogeneities, all CT replacement methods that were evaluated required manual segmentation of bone. Manual bone segmentation is time-consuming, making its clinical implementation impractical. Currently, different avenues for automatically segmenting bone in MR images of the prostate are under investigation. Both atlas-based [8] and statistical model-based approaches [9, 10] have been proposed. In atlas-based methods, probabilistic methods are employed to automatically segment structures of interest. Atlas-based methods rely on the accuracy of the implemented multimodal deformable image registration algorithm and, therefore, appear to be prone to the same MR-CT registration errors that implementation of an MR-only treatment planning workflow attempts to avoid. Statistical methods utilize magnitude information from several MR sequences to identify clusters associated with different segment types in the joint histogram and to determine the probability that a voxel belongs to each of the clusters. Like air, bone appears as a signal void in conventional MR images because of its low proton density and extremely short T2 signal lifetime [19]. Therefore, bone tends to be segmented with air. While ultrashort echo time (UTE) sequences may help to obtain a discernible signal from bone in order to separate bone from air, UTE has mainly been focused on the head region because of difficulties in attaining acceptable image quality at larger FOVs.

While reducing the time required for image post-processing and synCT generation is important, it is also important to reduce the scan time for the patient. The stated time to acquire the requisite images for our synCT workflow is ~18 min for a 1.0 T scanner. By contrast, a CT-SIM scan can be obtained in <1 min. Long scan times may cause degradation of image quality due to patient motion or variation in patient internal anatomy (e.g. changes in bladder and rectal filling). Scan times would be reduced at higher field strengths where increases in signal-to-noise ratio would also enable more aggressive acceleration factors, thereby reducing acquisition time. Future work will explore reducing the number of image sets needed to generate synCTs without introducing significant errors.

Variation in the level of rectal and bladder filling has been shown to deform the shape and to shift the position of the prostate and seminal vesicles by as much as 0.7 cm [20], leading to a reduction in local control for prostate cancer radiotherapy [21]. Therefore, the level of bladder and rectal filling must be reproducible between simulation and delivery of treatment. There is an increased risk that the level of rectal and bladder filling may change during an MR session simply due to the extended scan time for MR compared to that of CT. To mitigate this risk, it was recommended that steps be taken to ensure the patient has a full bladder [22] and empty rectum [21] prior to simulation such as regular patient instruction before and during the course of treatment. In extreme cases, administration of an enema prior to simulation may be warranted [21].

Results from Patient 11 indicate that synCT may provide greater value in regions with a higher air-tissue ratio, such as the brain or head and neck where airways are more prevalent. Manual segmentation in these regions is more difficult and time-intensive than for the prostate and would only further strain clinical workflow. For future implementations of the synCT workflow in the brain to be viable clinically, some method to automatically and accurately differentiate air from bone would be necessary. A promising method for accurately segmenting air separately from bone using UTE phase information is currently under development in our group.

## Conclusions

Overall, synCT-based treatment plans provided dosimetric and gamma analysis values in close agreement to original CT-based treatments plans consistent with previously published bulk density assignment-based substitute CT images, with significant improvement relative to those other substitute CT images observed for bladder.
